# Stability Analysis of a Run-of-River Diversion Hydropower Plant with Surge Tank and Spillway in the Head Pond

**DOI:** 10.1155/2014/874060

**Published:** 2014-10-14

**Authors:** José Ignacio Sarasúa, Paz Elías, Guillermo Martínez-Lucas, Juan Ignacio Pérez-Díaz, José Román Wilhelmi, José Ángel Sánchez

**Affiliations:** Department of Hydraulic, Energy and Environmental Engineering, Technical University of Madrid, 28040 Madrid, Spain

## Abstract

Run-of-river hydropower plants usually lack significant storage capacity; therefore, the more adequate control strategy would consist of keeping a constant water level at the intake pond in order to harness the maximum amount of energy from the river flow or to reduce the surface flooded in the head pond. In this paper, a standard PI control system of a run-of-river diversion hydropower plant with surge tank and a spillway in the head pond that evacuates part of the river flow plant is studied. A stability analysis based on the Routh-Hurwitz criterion is carried out and a practical criterion for tuning the gains of the PI controller is proposed. Conclusions about the head pond and surge tank areas are drawn from the stability analysis. Finally, this criterion is applied to a real hydropower plant in design state; the importance of considering the spillway dimensions and turbine characteristic curves for adequate tuning of the controller gains is highlighted.

## 1. Introduction

Nowadays, the interest in run-of-river hydropower plants is increasing. Climate change, shortage of appropriate places to build conventional hydropower plants, or generating electricity as near as possible to the consumption site (distributed generation) are some reasons for considering this type of plants.

There is an increasing need to develop clean energy technologies to cope with the problems relating to climate change, sustainable development, and energy security. Although hydropower is currently the principal renewable electricity generation source, its development would require overcoming some barriers concerning environment, public acceptance, and economic aspects [1].

In some regions, such as Asia Pacific and Central and South America, hydropower capacity is expected to increase significantly along next years. For instance, in Ecuador, hydropower produced more than one half of the generated electrical energy in 2012 [2], and one of the objectives of the “Plan Maestro de Electrificación” (Electrification Master Plan) 2012–2021 is that 90% of electric energy is to be generated from renewable sources, hydro and wind. However, in other countries, expected hydropower capacity increase would be lower than planned for other renewables technologies; in Spain, planned hydropower development is mainly based on run-of-river hydro plants, small hydro units for harnessing the energy associated with minimum environmental flows and refurbishment of existing plants [3].

Run-of-river hydro plants have become more important in recent years. Such plants are characterized by the small or zero storage capacity of the head pond and therefore the generated energy depends to a great extent on the available flow in the river. The interest in this type of plants is due to the effect of several factors.

- Run-of-river hydro plants allow harnessing the energy associated with water flows for other uses, such as water supply or irrigation, or the environmental minimum flows.
- In most cases, conventional hydro plants with reservoir give rise to significant environmental effects, such as physical barriers for fish movements or sediments transport along the river [4], visual impact, flooded areas, and land use issues.
- Size of run-of-river hydro plants is usually limited; therefore, these plants are connected to distribution networks and contribute to the development of distributed generation. In addition, initial and operating costs are lower than those associated with other renewables sources, such as wind parks [5].

Run-of-river plants have limited regulation capacity and in general do not participate in power-frequency regulation. However, some ways of participation of run-of-river hydro plants in this task have been proposed. The joint operation of several small hydro plants for providing primary, secondary, and tertiary regulation services is analyzed in [4]. The isolated operation of small hydro plants in remote areas is considered in [5]; the power-frequency regulation would be provided by changing the turbine speed, when the discharged flow through the turbine is lower than the available river flow; thus, variable-speed generation equipment is required.

The most frequently used control scheme in run-of-river plants is based on controlling the water level in the head pond [6–13]. Water level control has the advantages of its simplicity and robustness [8], allows minimizing the flooded area [13], and is compatible with other uses of spillway requiring constant water level. In small head schemes, as considered in [6], it is important to maintain the water level within strict limits. Different algorithms for controlling the water levels in three or more cascade hydro plants are presented in [7, 12]. In [10] the use of a dead band for minimizing the regulator movements is analyzed. The control system proposed in [14] is aimed at maintaining a minimum environmental flow in the intermediate river reach between the intake and the tailrace.

PID regulator has been extensively used in turbine governors of conventional hydro plants. The adjustment of the PID gains has been studied by several authors aiming to obtain a good dynamic response; among the first contributions, it is noteworthy to mention the works by Hovey and Paynter [15, 16]; in [17], the Routh-Hurwitz criterion is applied to define the stability region. A state variable model is used in [18, 19] for analyzing the influence of PI gains on the eigenvalues of the dynamic matrix. The root-locus technique has also been applied to the adjustment of PID gains [20, 21]. Although in more recent works advanced control techniques have been applied [22], the interest for robust PID controllers is still active [23]. In [24], the PID gains are analytically determined by pole placement; good performance was obtained in simulations and field tests conducted in a real plant. In [25, 26], the stability study and the tuning of the PID were carried out by eigenvalues analysis; oscillation modes were identified and associated with the different elements of the power plant.

In the case of water level control in [8], it is found that with a PI controller a good response may be obtained; the derivative component could be affected by the noise transmitted with the sensor signal. The root-locus technique is used to tune the controller in [12]. In [9], the stability regions are determined in terms of some design parameters; the tuning of the PI controller is based on a heuristic criterion derived from the root-locus plot. The influence of the operating point in the dynamics response is studied, concluding that for better performance the controller gains should be adapted to the operation conditions.

The aim of this paper is the study of the stability of the water level control system of a run-of-river hydropower plant under normal operating conditions. Then, a small perturbation analysis will be used. Two different operation modes are studied. In normal hydrologic conditions, the water level control system operates in a conventional way: the wicket gates position is changed, adapting the discharged flow to the inflow, in order to maintain the water level close to the reference value. In flooding operating conditions, the excess flow is discharged through a spillway; in this case, the water level reference is changed in order to allow controlling the spilled flow as in [14]. The dynamics of this operation mode has substantial differences with respect to the normal operation mode [8] and requires a specific analysis. Additionally, a diversion plant with surge tank is considered in order to extend the applicability of the results; in this configuration, the stability of level control should be studied with more detail due to the higher order of the involved dynamics. Following the methodology described in [9, 13], the stability regions are determined and the PI gains adjusted.

The results obtained in this study have been applied to Ocaña II hydro plant (Cuenca, Ecuador) which is currently in design phase.

The paper is organized as follows. In Section 2, the plant dynamic model used in the study is described. In Section 3, a small perturbation stability analysis is discussed and in Section 4 the PI controller is tuned by using a heuristic criterion. The theoretical results are applied to the case study in Section 5, verifying the adequacy of the proposed tuning criterion. Finally in Section 6 the conclusions derived from this study are presented.

## 2. Modeling

**Figure 1:**
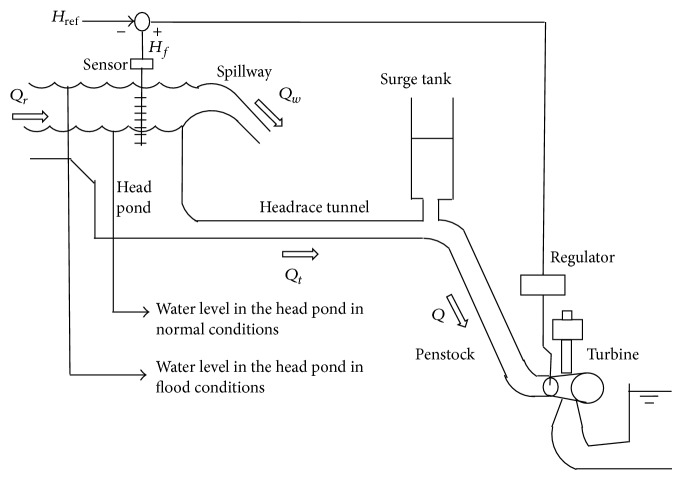
Run-of-river diversion hydropower plant with surge tank and spillway in the head pond.

In this section, the dynamic model of a run-of-river diversion hydropower station with pressurized conduits and surge tank is described. The head pond, where the intake is located, is created by a small dam with a spillway that allows evacuating the excess flow when the plant operates under flood conditions. The water surface in the head pond in normal operation is below the spillway level. A sensor level is placed near the intake and its signal is sent to the governor, which adjusts the wicket gates position in order to keep the water level constant in both situations: normal or flood. The plant layout can be seen in Figure 1.

**Figure 2:**
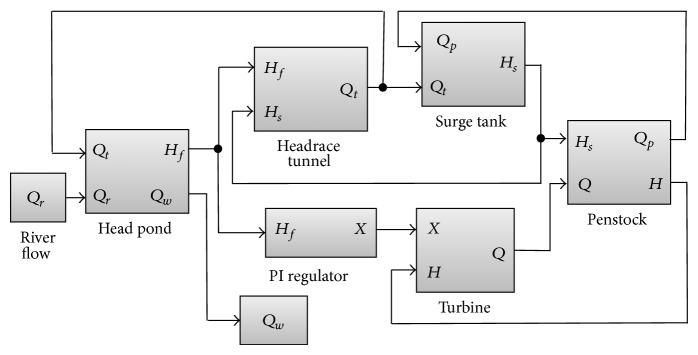
Block diagram of the control system.

The block diagram of the power plant dynamic model, comprising the hydraulic system and the PI regulator, can be seen in Figure 2. Main components of the model are described below.

The power plant modeled is supposed to operate connected to a large network. Therefore, the unit speed variations are not relevant and are damped in a very short time interval compared to the time scale of interest in this study [9, 27]; thus, speed may be supposed to remain constant at its synchronous value.

### 2.1. Hydraulic System

The elements that define the hydraulic system are the head pond, the headrace tunnel, the surge tank, the penstock, and the turbine. The dynamics of each component is expressed by different equations as presented below. The notation used throughout the paper is defined in Appendix B.

*Head Pond.* Consider

(1) $$\begin{array}{c} A_{f}\frac{{dH}_{f}}{dt}=Q_{r}-Q_{w}-Q_{t}, \end{array}$$

where *Q*_*w*_ is the flow through the spillway. It is equal to zero in normal operation. When the plant is working under flood conditions, the flow is calculated with the expression

(2) $$\begin{array}{c} Q_{w}=C_{d}L_{\text{aliv}}\sqrt{{\left(Z_{\text{ref}}-Z_{\text{aliv}}\right)}^{3}}. \end{array}$$

*Headrace Conduit.* The equations that describe transient-state flows in close conduits are mass and momentum conservation expressions [28]. A lumped parameters model has been used to represent the headrace conduit behavior [27, 29, 30].

**Figure 3:**
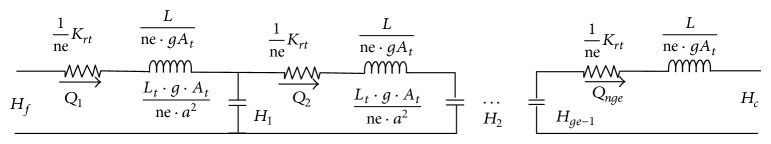
Scheme of the headrace tunnel model.

The system of ordinary equations obtained can be represented as a series of Γ-shaped consecutive elements. The “orientation” of the Γ-shaped elements may vary according to the upstream and downstream boundary conditions of the pipe. A scheme of the model can be seen in Figure 3. In this case, these boundary conditions are given by the head at both ends of the conduit.

The set of expressions applied to each element are

(3) $$\begin{array}{c} \frac{L}{\text{ne}\cdot gA_{t}}\frac{dQ_{i}}{dt}+\frac{1}{\text{ne}}K_{rt}\left|Q_{t}\right|Q_{t}=H_{i-1}-H_{i},\\ \frac{\text{ne}\cdot a^{2}}{L_{t}\cdot g\cdot A_{t}}\frac{dH_{i}}{dt}=Q_{i}-Q_{i+1}, \end{array}$$

where ne is the number of Γ-shaped elements the pipe is divided in.

*Surge Tank.* Consider

(4) $$\begin{array}{c} A_{s}\frac{{dH}_{s}}{dt}{=Q}_{t}-Q. \end{array}$$

*Penstock.* The conduit that joins the surge tank and the turbine is modeled as the headrace tunnel using the lumped parameters approach.

*Turbine.* The aim of the model is to study transients slower than the turbine response. In these cases [9, 31], a static model is used to include the turbine dynamics (5). The power plant modeled has two identical units, which are supposed to work at the same operating point; thus, a single equivalent turbine has been considered:

(5) $$\begin{array}{c} Q=f_{Q}\left(H,X\right). \end{array}$$

**Figure 4:**
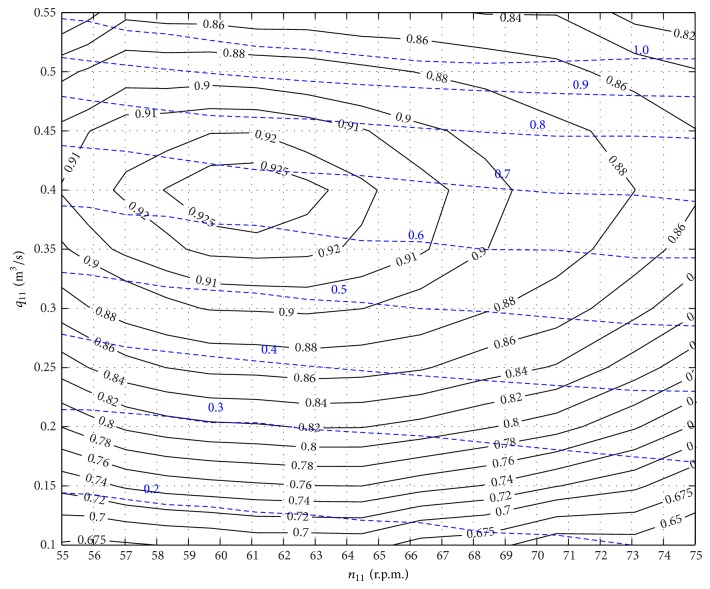
Hill curves.

Equation (5) should be obtained from the turbine hill curves; in Figure 4, the efficiency hill of the turbine considered in the case study is shown.

### 2.2. PI Regulator

A PI regulator modifies the wicket gate opening in order to maintain a constant water level in the head pond. The dynamics of this regulator can be expressed by

(6) $$\begin{array}{c} X=K_{p}\left(H_{f}\left(t\right)-H_{\text{ref}}\right)+K_{i}\overset{t}{\underset{0}{\int }}\left(H_{f}\left(t\right)-H_{\text{ref}}\right)dt_{\mathrm{int}}, \end{array}$$

where *K*_*p*_ is the proportional gain and *K*_*i*_ is the integral gain.

## 3. Stability Analysis

### 3.1. Linearized Model

In order to study the system stability following the Routh-Hurwitz criterion, a linear model should be used. Some assumptions have been done in order to linearize the above presented equations.

(i) Penstock dynamics is not considered because the associated time constant is in most cases very small compared to the relatively slow dynamics of the other components [32]. In the considered plant configuration, friction losses are mainly due to the headrace conduit [9] and therefore friction losses in penstock can also be neglected.
(ii) The oscillations of water level in the head pond are much slower than pressure waves in the conduits. Then, the headrace tunnel dynamics is approximated by a rigid water column model (7). The complete model described in Section 2.1 will be used only in the simulations:
(7) $$\begin{array}{c} \frac{L_{t}}{gA_{t}}\frac{dQ_{t}}{dt}+K_{rt}\left|Q_{t}\right|Q_{t}=H_{f}-H_{s}. \end{array}$$

In order to obtain the state equations the controller equation is expressed in the form

(8) $$\begin{array}{c} \frac{dX}{dt}=K_{i}\left(H_{f}-H_{\text{ref}}\right)+K_{p}\frac{d\left(H_{f}-H_{\text{ref}}\right)}{dt}. \end{array}$$

Previous equations have been expressed in per unit values and linearized around an initial equilibrium point; the obtained equations are included in Appendix A. The resulting linear model, in state space form, is

(9) dXdt=A·X+B·U,

(10) X=[qthfhsτ];  U=[qrhrefnt],

(11) B=[0001Tf0000−b12TskpTf−ki0],

(12) A=[−2pqt0Tw1Tw−1Tw0−1Tf−32M001Ts0−b11Ts−b13Ts−kpTfki−kp32M00],

where the parameter *M* (13) has been introduced to consider the effect of spillway dimensions

(13) $$\begin{array}{c} M=\frac{C_{d}L_{\text{aliv}}}{A_{f}}\sqrt{Z_{\text{ref}}-Z_{\text{aliv}}}. \end{array}$$

### 3.2. Stability Analysis

The characteristic polynomial of the matrix **A** may be expressed as

(14) $$\begin{array}{c} P\left(A\right)=\lambda^{4}+a_{1}\lambda^{3}+a_{2}\lambda^{2}+a_{3}\lambda +a_{4}, \end{array}$$

where the coefficients are

(15) a1=2pqt0Tw+b11Ts+32M,a2=1+2pqt0b11TsTw+322pqt0Ts+b11TwTsTwM+1TfTw,a3=321+2pqt0b11TsTwM+b13kp+b11TsTwTf,a4=kib13TsTwTf.

According to Routh-Hurwitz criterion, the system (9) is asymptotically stable if the following conditions are satisfied. (a) All coefficients of the characteristic polynomial (14) must be different from zero and of the same sign; (b) the elements of the first column of the Routh array must be positive. In practice, the first condition is always fulfilled, and the second condition may be shown to reduce to

(16) $$\begin{array}{c} a_{1}a_{2}a_{3}-{a_{3}}^{2}-{a_{1}}^{2}a_{4}>0. \end{array}$$

In order to study the influence of the surge tank and head pond dimensions in the stability of the power plant, the following parameters *n* and *m* are defined, as described in [9, 13]:

(17) $$\begin{array}{c} n=\frac{A_{s}}{A_{\text{th}}},\;\;m=\frac{A_{f}}{A_{s}}, \end{array}$$

where *A*_th_ is the surge tank cross-sectional area that guarantees stability according to the Thoma criterion [13, 33]. Although this widely known criterion was formulated for load-frequency control, it provides an interesting reference value for the present study. The following expression (18) gives its value as a function of the plant design parameters:

(18) $$\begin{array}{c} A_{s}>A_{\text{th}}=\frac{L_{t}Q_{b}^{2}}{2gH_{b}^{2}A_{t}p}=\frac{Q_{b}}{2H_{b}p}T_{w}. \end{array}$$

Introducing *n*, *m*, and *M* parameters, stability condition results in

(19) ki<{(qt0n+b11+32MnTw2p)×[(1+2pqt0b11)m+(qt0n+b11)m23MTw+1]−(1+2pqt0b11)mn2pTw32M+(b13kp+b11)}·(1+2pqt0b11)(mn/2p)Tw(3/2)M+b13kp+b11(qt0n+b11+(n/2p)Tw(3/2)M)2b13mTw,

where *k*_*i*_ and *k*_*p*_ are the controller gains in p.u.

**Figure 5:**
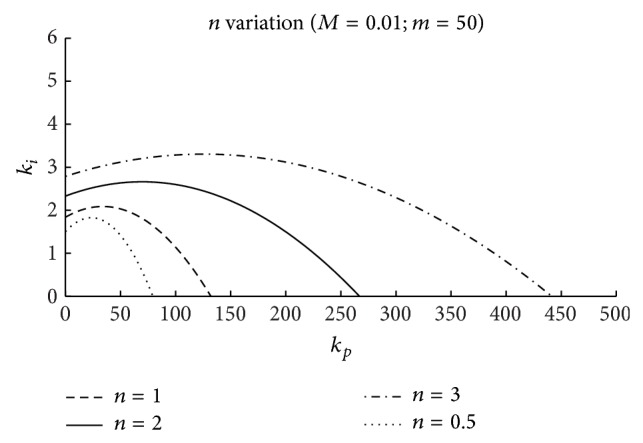
Stability regions: effect of the surface area of the surge tank (*n*).

**Figure 6:**
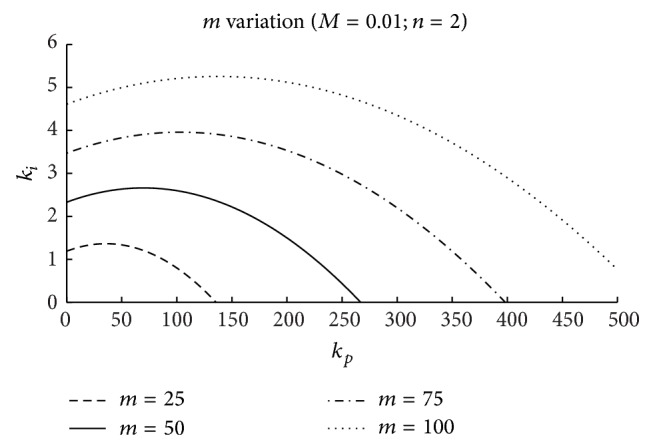
Stability regions: effect of the surface area of the head pond (*m*).

**Figure 7:**
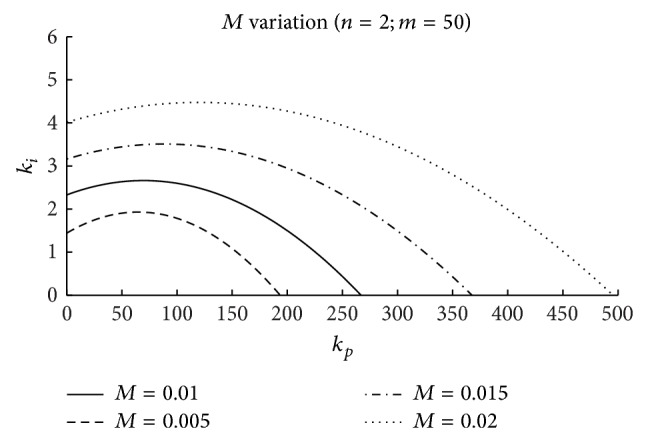
Stability regions: effect of the spillway dimension (*M*).

Figures 5, 6, and 7 include the effects of the surge tank cross-section area (*n*), the head pond surface area (*m*), and the spillway parameter (*M*), respectively, in the stability region. The coefficients *b*_11_ and *b*_13_, acquired from the hill curves, and the initial per unit headrace conduit flow variable, *q*_*t*_^0^, used to obtain these figures, correspond to the initial operating point; the per unit initial flow and head losses in the conduit *p* have been assumed to be equal to 1 and 0.08 p.u., respectively; for the water inertia time constant, *T*_*w*_, the value 12.09 s has been used, as in the case study.

As it can be seen in these figures, the magnitude of the controller gains is large. This is due to the very small value of the error signal (p.u. level variation in head pond). Thus, for a practical implementation of this control scheme, a signal conditioner could be required.

In the three figures, the area under the curves determines the stable region, that is, the combination of *k*_*p*_ and *k*_*i*_ that guarantees a stable operation of the level control system. As it seems intuitively logical, it can be observed that in the three cases the system stability region is extended when parameters *n*, *m*, and *M* increase.

In Figure 5, the effect of varying the *n* parameter is represented. In the figure, it can be seen that for *n* values below 1.0 there exists a stability region. This fact indicates that in the case of level control the fulfillment of the Thoma condition is not necessary for system stability [9].

In Figure 6, the effects of varying *m* are considered. The figure shows that this parameter influences notably the system stability.

The characteristics of the spillway (length, spillway discharge coefficient, etc.), reflected in the parameter *M* (13), affect the stability of the plant, as can be seen in Figure 7. Therefore, the design of the spillway should be taken into account if water level control is considered.

In order to compare the stability regions in both operation modes (normal and flood), (19) is simplified using *M* = 0. The resulting expression (20) was developed in [9]:

(20) ki<{(qt0n+b11)[(1+2pqt0b11)m+1]−(b13kp+b11)}×(b13kp+b11)(qt0n+b11)2b13m.

**Figure 8:**
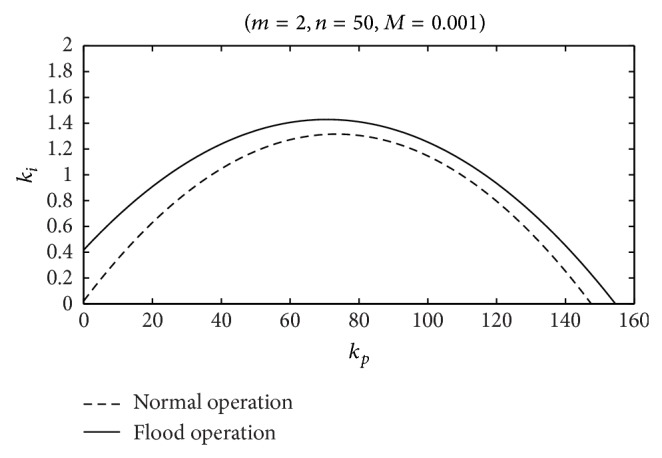
Stability regions: comparison of normal and flood operation.

As it is shown in Figure 8, the stability region, when a part of the river's flow is evacuated by the spillway, is bigger than the region obtained in normal operation. *M* = 0.010 is equivalent to a spillway with a length of 7.5 m in a head pond of 900 m^2^ and considering 0.5 m for the height of water level above the spillway. Therefore, it can be stated that the change in the head pond dynamics produced by the spillway contributes positively to the stability of the water level control. Admittedly, in flood operation mode head pond surface area (*A*_*f*_) may be affected for the change in the reference water level. However, the influence in the stability regions due to this change is quite small; so it will be neglected.

In (19) and (20), it can be seen that turbine operating conditions are another important factor for stability. The initial per unit headrace conduit flow, *q*_*t*_^0^, and the turbine coefficients, *b*_*ij*_, change when the plant is operating with partial load different from rated conditions. This fact affects the stability boundaries as well as the results of applying the tuning criterion of the PI gains proposed in the next section. This issue is studied in detail in [9], so in this paper the work focuses on the influence of the spillway on the stability and control of a run-of-river power plant.

## 4. PI Gains Tuning

The characteristic polynomial (13) of the linearized system has, in general, four conjugate complex roots:

(21) $$\begin{array}{c} p_{12}=a\pm jb,\;\;p_{34}=c\pm jd. \end{array}$$

Other authors have worked using the pole placement for tuning the governor gains. For example, in [34] some strategies for obtaining an appropriate response were proposed but in the context of load-frequency control. In [35], water level of three coupled tanks is controlled by a structure of PI controllers; PI controllers were tuned using pole placement method by reducing the overshoot and the settling time. In this paper, one of the main priorities is to minimize the effort made by the servo which acts on the turbine wicket gates following the philosophy expressed in [10] and reducing the settling time. For these purposes, the criteria proposed in [9] are used in this paper and summarized below.

1. The real part of each pair of conjugated poles should have approximately the same value so that the settling times are similar and the appearance of a slow pole is avoided.
2. One of these pairs should have its imaginary part close to zero, thus avoiding the appearance of oscillations as far as possible.

Analytical expressions (22) for the proportional gain in p.u. *k*_*p*_ and the integral term in p.u. *k*_*i*_ are obtained from the rules formulated. As stated in [9], it is worthy to mention that these expressions are independent of each other.

**Figure 9:**
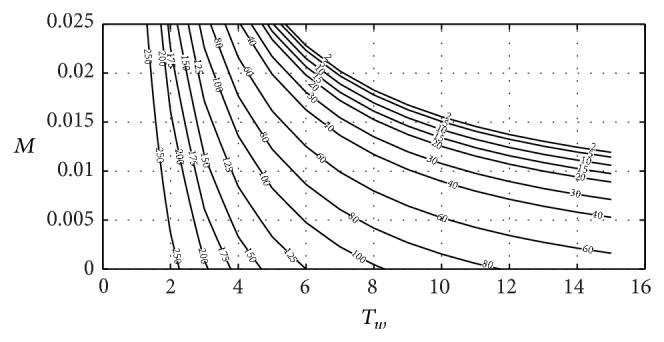
Relation between *M*, *T*_*w*_ and *k*_*p*_.

**Figure 10:**
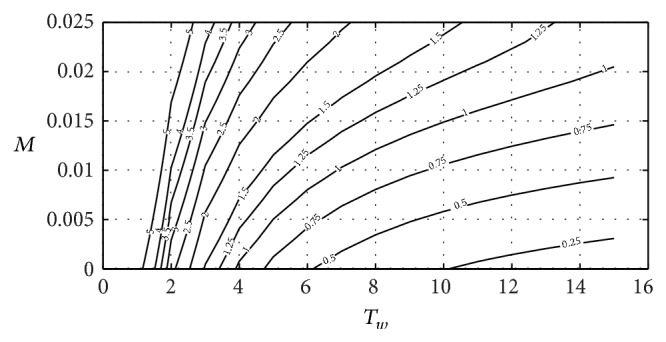
Relation between *M*, *T*_*w*_, and *k*_*i*_.

Figures 9 and 10 include the relation between *T*_*w*_ and *M* (spillway dimension) with the *k*_*p*_ and *k*_*i*_ parameters obtained using (22). It has been supposed that the surge tank surface is equal to the surface proposed by [33] for the surge tank dimensioned in Ocaña II power plant. A head pond surface area of 900 m^2^ will be assumed. This value corresponds to head pond surface area in Ocaña II power plant:

(22) kp=1b13{[TsTwTf2(2pqt0Tw+b11Ts+32M)×{1+2pqt0b11TwTs+322pqt0Ts+b11TwTwTsM+1TwTf−14(2pqt0Tw+b11Ts+32M)2}−32(1+2pqt0b11)TfM]−b11},ki=TsTwTf16b13×{1+2pqt0b11TwTs+322pqt0Ts+b11TwTwTsM+1TwTf−516(2pqt0Tw+b11Ts+32M)2}×(2pqt0Tw+b11Ts+32M)2.

## 5. Study Case

Ocaña II hydropower plant, located in Cañar (Ecuador), is a run-of-river diversion plant just downstream Ocaña I power plant which is operating nowadays in Cañar River. The plant consists of a head pond with a spillway for excess of flow during flood periods, long headrace conduit of more than 5 kilometers, surge tank, penstock, and two Francis turbines. The main features of the plant are reflected in Table 1.

The results of the previous stability analysis, as well as the followed PI tuning criterion, have been applied to a hydropower plant that is currently in design stage. The plant response has been obtained by means of simulations. The results obtained were useful in the design process and will be used for the implementation of the control system.

Although the model used for both the stability analysis and obtaining the mathematical expressions of the controller gains is a linear model with certain simplifications, the model used for simulations is the nonlinear one described in Section 2 and includes penstock dynamics, as well as nonlinearities associated with losses in the conduits so as to obtain results closer to the real plant response. The headrace conduit has been divided into six equal elements and the number of elements for the penstock is four.

### 5.1. Application of the Results of the Stability Analysis

**Figure 11:**
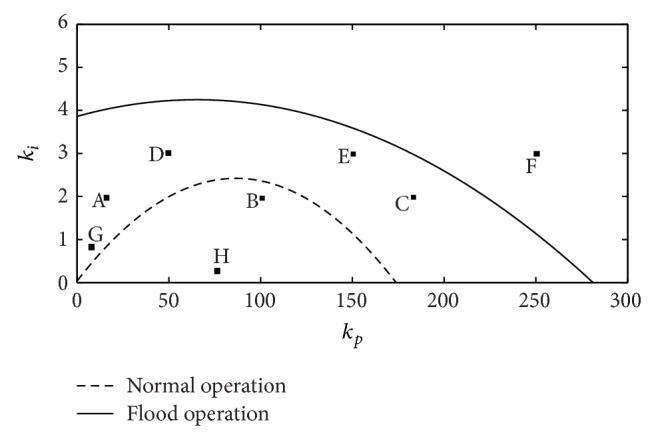
Stability regions in normal and flood operation conditions.

The stability regions obtained from (19) and (20), corresponding to the power plant under study for both operating modes, are shown in Figure 11. Table 1 contains the parameters needed to make the power plant characteristics adequate for the mathematical expressions.

Two cases are considered to evaluate the plant response in this situation. The first one is the case evaluated in [9] in which the reference water level in the head pond is below the spillway (normal condition). In the second case, the reference water level in the head pond is over the spillway (flood conditions). In both operating modes scenarios, the initial flow released through the turbines is 20 m^3^/s. In normal conditions, initial turbine(s) flow coincides with the river flow; under flood conditions, the river flow is 25.4 m^3^/s, the spillway evacuating 5.4 m^3^/s.

In both cases, the system response to a sudden reduction in river flow of 3 m^3^/s has been simulated for each pair of gains included in Table 2. Pairs of gains are highlighted in the stability regions (A–F) (Figure 11). In Table 2, it is indicated whether or not each pair of gains is located inside or outside the corresponding stability region.

**Figure 12:**
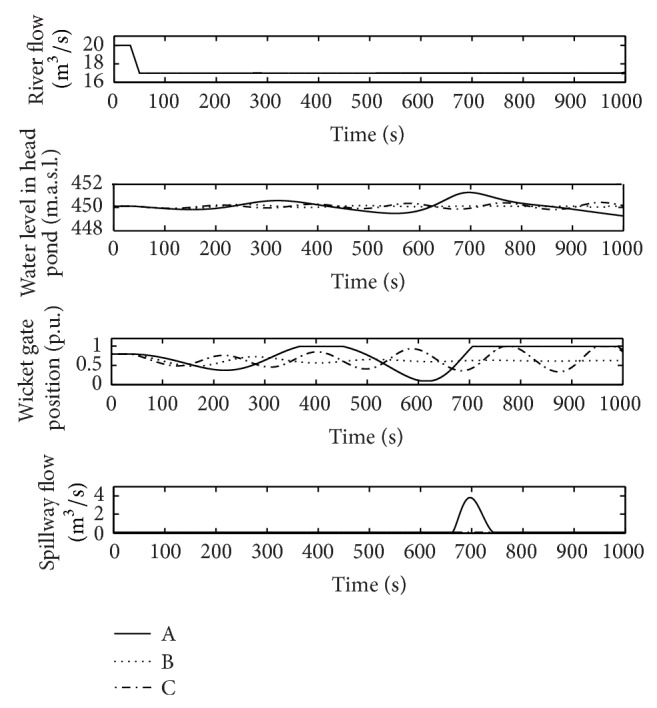
River flow, wicket gates position, water level in the head pond, and spillway flow, in normal operation (points A, B, and C).

**Figure 13:**
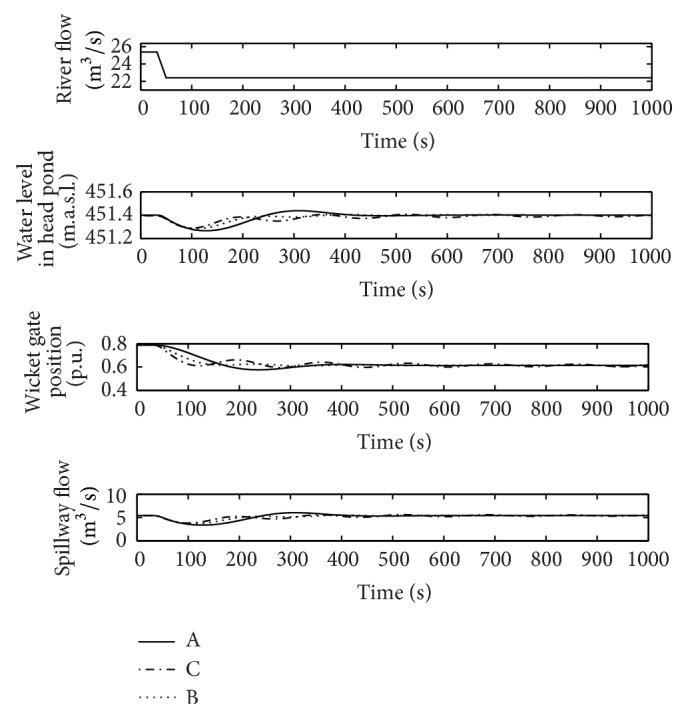
River flow, wicket gates position, water level in the head pond, and spillway flow, in flood operation (points A, B, and C).

Figure 12 shows the plant response with the PI tuned with the gains corresponding to A, B, and C in the case without spillway, in normal conditions. As can be seen in Figure 12, only in case B (*k*_*p*_ = 100, *k*_*i*_ = 2) the plant response is stable. This result is in agreement with the stability region depicted in Figure 11. In turn, Figure 13 shows the plant response under flood conditions, with spillway, in points A, B, and C. As it can be deduced from Figure 11, the plant response is stable with all pairs of gains (A, B, and C). The differences between the responses of the power plant are the consequence of the variation in the gains of the PI regulator. The damping of oscillations decreases for higher values of *k*_*p*_, but the amplitude of the oscillations is reduced in these cases. The minimum value of the controlled variable (water level in the head pond) is not as important as in the case of load frequency control, where frequency must be maintained within a severe interval. Therefore, low values for *k*_*p*_ could be appropriate for reducing the wear and tear of the servo mechanism that moves the wicket gates position.

**Figure 14:**
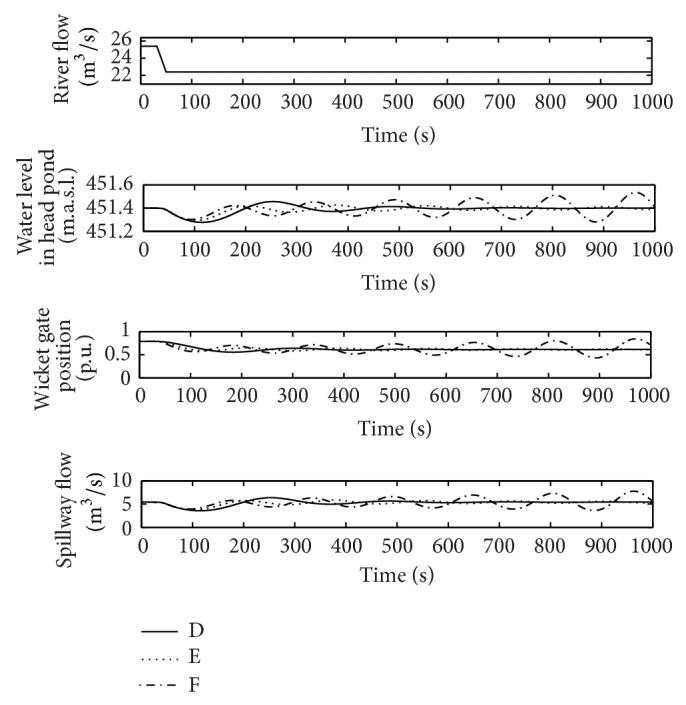
River flow, wicket gates position, water level in the head pond, and spillway flow, in flood operation (points D, E, and F).

Figure 14 shows the response in D, E, and F points in order to verify the validity of the stability region presented in Figure 11 for flood conditions. As expected, the response for adjustment at point F results unstable. The simulations have been carried out only with the plant in flood conditions because all the responses for points D, E, and F are unstable when the water level is below the spillway in the head pond.

### 5.2. Application of the PI Tuning Criterion

The expressions (22) are applied for tuning PI gains of Ocaña II power plant both in normal (*M* = 0) and in flood operating conditions.  Table 3 contains the numerical values of the gains in both situations and in Figure 11 the two pairs of gains are positioned in the corresponding stability regions.

**Figure 15:**
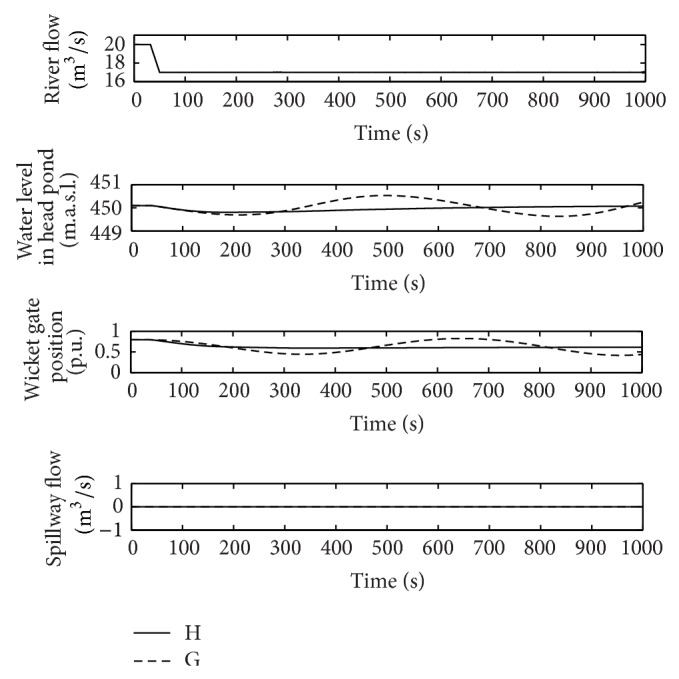
River flow, wicket gates position, water level in the head pond, and spillway flow, points G and H in normal operation.

From Figure 11, it can be deduced that the pair of gains for flood operation (G) can cause instabilities if used during normal operation. In order to support this idea, the same sudden reduction of 3 m^3^/s in the river flow has been simulated in normal conditions with pairs of gains G and H. The response of the power plant is shown in Figure 15.

The instability observed for the pair of gains G confirms the results obtained from the linear model. Moreover, as it can be seen in Figure 15, when the controller is tuned according to the criterion (gains H), the oscillation of the wicket gates position is almost eliminated, thus contributing to both increasing the equipment service life and reducing the settling time of the response.

**Figure 16:**
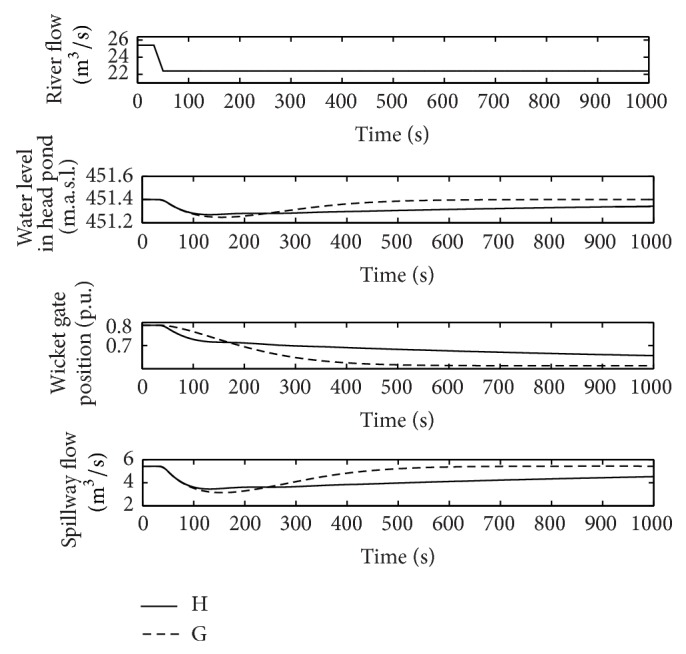
River flow, wicket gates position, water level in the head pond, and spillway flow, points G and H in flood operation.

From Figure 11, it can be deduced that, unlike the previous case, using the pair of gains obtained for normal operation (H) should not give rise to instabilities in flood conditions. Nevertheless, as it is shown in Figure 16, the quality of the power plant response is considerably worse than the one obtained with the pair of gains specific to flood conditions (G).

The results obtained in the case study highlight the importance of carrying out a stability analysis similar to the one presented here in the design phase of a run-of-river diversion power plant. The results of the stability analysis can be of much help for making decisions about certain design parameters of the power plant, as well as about the gains of the water level controller.

## 6. Conclusions

In this paper, the stability of a run-of-river diversion hydropower plant with a spillway in the head pond that evacuates a portion of the river flow is analyzed. A PI controller is used for maintaining a constant water level in the head pond; the effects of a surge tank have been considered. For this purpose, a small perturbation stability analysis based on the Routh-Hurwitz criterion has been carried out. From the stability analysis, it has been demonstrated that the existence of a spillway in the analysis improves the stability of the plant water level control; when the dynamics of the spillway is included (flood operation mode), the stability region covers a broader area than in normal operation mode (without spillage). On the other hand, it has been found that the fulfillment of the well-known Thoma stability condition is not necessary in this case.

In addition, analytical expressions, based on a heuristic criterion, have been obtained for both the proportional and integral gains in flood operation conditions. It is worth noting that the said expressions depend on the dimensions and flow evacuated by the spillway, the initial per unit headrace conduit flow, and the turbine parameters. Additionally, it is interesting to emphasize that these analytical expressions have been found to be independent of one another.

The stability analysis, as well as the PI tuning criterion, has been applied to a real hydropower plant, currently in the design stage; the plant response has been obtained by means of simulations with a more detailed model. The results obtained have been found to be useful for the design process and may be used in the implementation of the control system.

The results of the stability analysis and the simulations have demonstrated that the operation with a reference water level above the spillway crest level (i.e., with a constant flow rate released through the spillway) is more stable than the one with a reference water level below the spillway crest level. Also, it has been found that the level controller gains should be updated in real-time operation, not only as a function of the actual operating point (full or partial load) but also as a function of the actual operating mode (normal or flood conditions) in order to guarantee a stable and quality response of the water level control system. Should the real-time gains updating not be possible, a similar analysis to the one carried out in this paper should be done to select a pair of gains that guarantees a stable response in as many foreseen operating points and modes as possible, with a reasonable response quality.

## Figures and Tables

**Table 1:** Plant dimensions, parameters and base values.

Element	Parameter		Value
Base values	Base head	*H*_*b*_	165.19 m
	Base flow	*Q*_*b*_	20 m^3^/s

Head pond	Horizontal section	*A*_*f*_	900 m^2^
	Spillway length	*L*_aliv_	9.00 m
	Spillway level	*Z*_aliv_	450.90 masl
	Water level reference under normal conditions	*Z*_ref_	450.10 masl
	Water level reference under flood conditions	*Z*_ref_	451.40 masl
	*M*		0.012

Head race conduit	Length	*L*_*t*_	5201 m
	Section	*A*_*t*_	5.31 m^2^
	Losses	Δ*H*_*t*_	13.21 m
	Losses (p.u.)	*p*	0.080 p.u.
	Time constant	*T*_*w*_	12.09 s
	Initial head-race conduit flow	*q*_*t*_^0^	1.00 p.u.

Surge tank	Section	*A*_*s*_	9.62 m^2^
	Thoma section	*A*_th_	9.15 m^2^
	*m*		1.052
	*n*		93.54

Penstock	Length	*L*_*p*_	801.15 m
	Section	*A*_*p*_	5.31 m^2^
	Losses	Δ*H*_*p*_	3.45 m

Turbine	*b*_11_		0.596
	*b*_13_		0.978

**Table 2:** Pairs of gains selected for carrying out simulations.

Point	*k*_*p*_	*k*_*i*_	Stability	
			Normal operation	Flood operation
A	20	2	NO	YES
B	100	2	YES	YES
C	180	2	NO	YES
D	50	3	NO	YES
E	150	3	NO	YES
F	250	3	NO	NO

**Table 3:** Pairs of gains from the tuning criterion.

Point	*k*_*p*_	*k*_*i*_
G-flood operation	11.96	0.724
H-normal operation	78.44	0.200

## Appendices

### A. Linearization

The equations that describe the behavior of the components of the power station have been linearized around an operating point as shown below. Per unit values have been extensively used. The following linearized expressions are obtained.

Head pond is

(A.1)
dhfdt=-1Tfqt-32Mhf+1Tfqr.

Headrace conduit is

(A.2)
dqdt=-2pqt0Twqt-1Twhf-1Twhs.

Surge tank-penstock-turbine is

(A.3)
dhsdt=1Tsqt-b11Tshs-b13Tsτ-b12Tsnt.

PI regulator is

(A.4)
dτdt=-kpTfqt+(ki-ki32M)hf-kihref-kpTfqr.

Turbine is

(A.5)
q=b11h+b12nt+b13τ,

where

(A.6)
Tw=LtQbgAtHb,Ts=AsHbQb,Tf=AfHbQb,p=KrtQb2Hb.

### B. Used Symbols

a : Wave celerity in the conduit (m/s)
Af : Head pond cross-sectional area (m^2^)
As : Surge tank cross-sectional area (m^2^)
At : Headrace conduit cross-sectional area (m^2^)
Ath : Minimum surge tank cross-sectional area according to Thoma criterion (m^2^)
Ap : Penstock cross-sectional area (m^2^)
bij : Parameters of the linearized turbine equations
Cd : Spillway coefficient
g : Gravity acceleration (m/s^2^)
H : Net head (m)
Hb : Base head (m)
Hf : Gross head in the head pond with respect to tailwater level (m)
hf : Variation of the gross head in the head pond (p.u.)
Hi : Head at the end of the i th Γ element of the conduit (m)
Href : Reference height in the head pond with respect to tailwater level (m)
href : Variation of the reference height in the head pond (p.u.)
Hs : Surge tank level with respect to tailwater level (m)
hs : Surge tank water level variation (p.u.)
Kp : Proportional gain of PI controller
kp : Proportional gain of PI controller (p.u.)
Ki : Integral gain of PI controller
ki : Integral gain of PI controller (p.u.)
Krt : Headrace loss coefficient
Lt : Headrace conduit length (m)
Laliv : Crest spillway length (m)
Lp : Penstock length (m)
nt : Turbine rotational speed (p.u.)
p : Headrace conduit losses (p.u.)
q : Turbine flow (p.u.)
Q : Turbine flow (m^3^/s)
Qb : Base flow (m^3^/s)
Qi : Flow in the i th Γ element of the conduit (m^3^/s)
Qr : River flow (m^3^/s)
qr : River flow variation (p.u.)
Qt : Headrace conduit flow (m^3^/s)
qt : Variation in headrace conduit flow (p.u.)
qt0 : Initial headrace conduit flow (p.u.)
Tf : Head pond time constant (s)
Ts : Surge tank time constant (s)
Tw : Headrace conduit time constant (s)
X : Wicket gates opening (mm)
Xb : Base value of the wicket gates opening (mm)
τ : Wicket gates opening variation (p.u.)
Zref : Reference height in the head pond (m.a.s.l.)
Zaliv : Crest spillway level (m.a.s.l.)
ΔHp : Friction losses in the penstock (m)
ΔHt : Friction losses in the headrace tunnel (m).
